# Comparative Analysis of Prognostic Potential of Pretreatment Blood-Based Biomarkers in Metastatic Bladder Cancer: Modified Glasgow Prognostic Score

**DOI:** 10.3390/jcm14061954

**Published:** 2025-03-13

**Authors:** Fatih Kus, Deniz Can Guven, Hasan Cagri Yildirim, Elvin Chalabiyev, Ilgin Koc, Omer Denizhan Tatar, Firat Sirvan, Yigit Berk Sahin, Ece Karaca, Furkan Kabukcu, Basar Alp Bay, Oguzalp Kavruk, Mustafa Erman

**Affiliations:** 1 Department of Medical Oncology, Faculty of Medicine, Hacettepe University, Ankara 06230, Turkeyermanm1968@gmail.com (M.E.); 2 Department of Internal Medicine, Faculty of Medicine, Hacettepe University, Ankara 06230, Turkey

**Keywords:** bladder, prognosis, pretreatment, biomarkers

## Abstract

**Background**: Metastatic bladder cancer (mBC) presents a significant global health challenge with a poor prognosis and considerably limited survival. Despite advancements in therapies, long-term survival remains difficult to predict. This study aimed to evaluate the prognostic potential of various pretreatment blood-based biomarkers, including the NLR, dNLR, LMR, PLR, SII, mGPS, CAR, AGR, PNI, PIV, and Bellmunt score, in mBC patients. **Methods**: A retrospective cohort of 133 patients from Hacettepe University Cancer Institute was analyzed. Kaplan–Meier survival analysis and Cox regression models were used to assess overall survival (OS) and progression-free survival (PFS). **Results**: There was a significant association between multiple biomarkers and OS in the univariate analysis, with a higher NLR, PLR, and SII linked to worse outcomes. However, in the multivariate analysis, only the modified Glasgow Prognostic Score (mGPS) maintained independent prognostic significance for OS (HR: 1.984, *p* = 0.013). This suggests that the mGPS, which reflects systemic inflammation and nutritional status, is a robust predictor of survival in mBC. **Conclusions**: This study highlights the potential of integrating blood-based biomarkers into clinical decision-making to improve personalized treatment strategies. However, prospective studies are needed to validate these findings and assess their applicability to newer therapies such as immune checkpoint inhibitors and antibody–drug conjugates.

## 1. Introduction

Metastatic bladder cancer (mBC) poses a substantial global health challenge, holding the ninth position in the list of the most commonly diagnosed cancers globally [1]. According to the Globocan data of 2020, bladder cancer was estimated to account for 573,278 new cases and to cause 212,536 deaths worldwide [2].

The prognosis for mBC is generally poor, with a median survival of approximately 15 months with systemic chemotherapy [3]. Despite recent advances in systemic therapy, including the emergence of immune checkpoint inhibitors and antibody–drug conjugates, the disease remains incurable [3]. However, the survival of bladder cancer patients varies considerably, and long-lasting responses and hence survival can be achieved in a subset of patients. Though several prognostic factors have been well established, additional prognostic factors may lead to better clinical decisions [4].

Blood-based inflammatory markers such as the neutrophil-to-lymphocyte ratio (NLR), lymphocyte-to-monocyte ratio (LMR), platelet-to-lymphocyte ratio (PLR), and systemic inflammation index (SII) have shown potential in predicting the prognosis of various cancers [5,6]. Over the past few years, an increasing number of studies have explored the prognostic value of various biomarkers in patients with mBC. Many of these studies have focused on the correlation between parameters such as the NLR, derived NLR (dNLR), LMR, PLR, SII, modified Glasgow Prognostic Score (mGPS), C-reactive protein-to-albumin ratio (CAR), albumin-to-globulin ratio (AGR), prognostic nutritional index (PNI), PIV (pan-immune inflammation value), and Bellmunt score with overall survival (OS) and progression-free survival (PFS) in bladder cancer patients [7,8,9,10,11,12,13]. However, most of these studies have focused on the pre- and post-operative measurements in patients undergoing radical cystectomy, while limited research is available specifically on mBC patients. Additionally, to the best of our knowledge, most studies focus on a single or a couple of indexes and no single study includes a comprehensive analysis of all these biomarkers in the context of mBC.

Therefore, this study aims to explore the prognostic value of hematological biomarkers in patients with mBC. We planned to analyze the associations between the NLR, dNLR, LMR, PLR, SII, mGPS, CAR, AGR, PNI, and OS in a retrospective cohort of patients diagnosed with mBC. The findings from this study could potentially contribute to the improvement of personalized treatment strategies.

## 2. Methods

This study included 133 patients diagnosed with metastatic breast cancer (mBC) who received treatment at Hacettepe University Cancer Institute between January 2010 and January 2023.

The inclusion criteria were as follows: a confirmed diagnosis of mBC, receipt of chemotherapy for mBC, availability of pre-chemotherapy laboratory data, and accessible records of treatment response, disease progression, and survival. Patients were excluded if pretreatment laboratory parameters were unavailable or if follow-up data were insufficient. Additionally, individuals with concurrent hematological or immunological disorders or those receiving immunosuppressive therapy for any reason were not included in the study.

### 2.1. Data Collection

Clinical and laboratory data were retrospectively extracted from electronic medical records. Collected information included baseline demographic characteristics, primary tumor features, metastatic sites, and various hematological biomarkers. The blood-based biomarkers analyzed in this study comprised the neutrophil-to-lymphocyte ratio (NLR), lymphocyte-to-monocyte ratio (LMR), platelet-to-lymphocyte ratio (PLR), systemic immune-inflammation index (SII), derived NLR (dNLR), C-reactive protein-to-albumin ratio (CAR), albumin-to-globulin ratio (AGR), prognostic nutritional index (PNI), and modified Glasgow Prognostic Score (mGPS). Additionally, confounding factors such as age, Eastern Cooperative Oncology Group (ECOG) performance status, and de novo metastatic status were recorded. Serum levels of selected biomarkers were monitored over time, and their prognostic potential was evaluated in relation to primary tumor characteristics, metastatic patterns, treatment modalities, and survival outcomes using appropriate statistical analyses.

### 2.2. Statistical Analysis

The required sample size was calculated using G*Power 3.1.9.4 software, considering the significance level and effect size of the established hypothesis.

Based on the hazard ratio (HR) reported by Nagai et al. (2021) [14], which indicated that an mGPS score of 2 in patients receiving second-line pemetrexed increased mortality risk by 2.06-fold (HR = 2.06, 95% CI: 1.37–3.11), the minimum sample size was determined. With α = 0.05, 1 − β = 0.95, and a test power of 95%, the required minimum sample size was calculated as 23 patients.

The prognostic significance of hematological biomarkers was assessed through Kaplan–Meier survival analysis and Cox proportional hazards regression models, evaluating their associations with overall survival (OS) and progression-free survival (PFS). OS was defined as the time from the initiation of chemotherapy for mBC to death from any cause, while PFS was defined as the time from chemotherapy initiation to disease progression or death from any cause.

Associations between hematological biomarkers and clinicopathological features were analyzed using chi-square tests or Fisher’s exact tests, as appropriate. A *p*-value < 0.05 was considered statistically significant.

## 3. Results

The mean age of the patients was 64.6 ± 0.9 years. Of the participants, 12.8% were female (n = 17), and 87.2% were male (n = 116). The majority of the patients had an ECOG performance status (PS) of 0 or 1 (n = 116, 87.9%), while the remaining patients had a PS of 2 or 3 (n = 16, 12.1%). The mean BMI was calculated to be 25.3 (SD: 4.48) (Table 1).

The blood test results showed the following mean values and standard deviations (SD): white blood cell (WBC) count was 8.75 ± 3.06 × 10^9^/L (range: 3.4–22.4 × 10^9^/L), hemoglobin (Hgb) concentration was 12.03 ± 2.12 g/dL (range: 7.2–16.4 g/dL), lymphocyte count was 1.67 ± 0.75 × 10^9^/L (range: 0.1–4.6 × 10^9^/L), neutrophil count was 6.10 ± 2.78 × 10^9^/L (range: 2.1–20.8 × 10^9^/L), monocyte count was 0.68 ± 0.27 × 10^9^/L (range: 0.1–1.65 × 10^9^/L), and platelet count was 296.49 ± 108.58 × 10^9^/L (range: 92–635 × 10^9^/L). The mean albumin and globulin levels were 3.76 ± 0.59 g/dL (range: 1.99–4.79 g/dL) and 3.26 ± 0.55 g/dL (range: 1.77–4.71 g/dL), respectively. The mean C-reactive protein (CRP) level was 6.84 ± 8.02 mg/L, with a range of 0.15–46.31 mg/L.

The median PFS of the study population was found to be 6.0 months (95% confidence interval (CI): 6.7–9.6), and the median OS was 10.5 months (95% CI: 12.8–18.6).

### 3.1. NLR

The median NLR was found to be 3.6 (0.46–64.0). Using this median as a cut-off, the median OS was 6.2 months (95% CI 4.3–8.1) in the higher NLR group and 20.3 months (95% CI 14.4–26.2) in the lower NLR group (HR: 2.17, 1.43–3.3 CI, *p* < 0.001).

**Figure 1:**
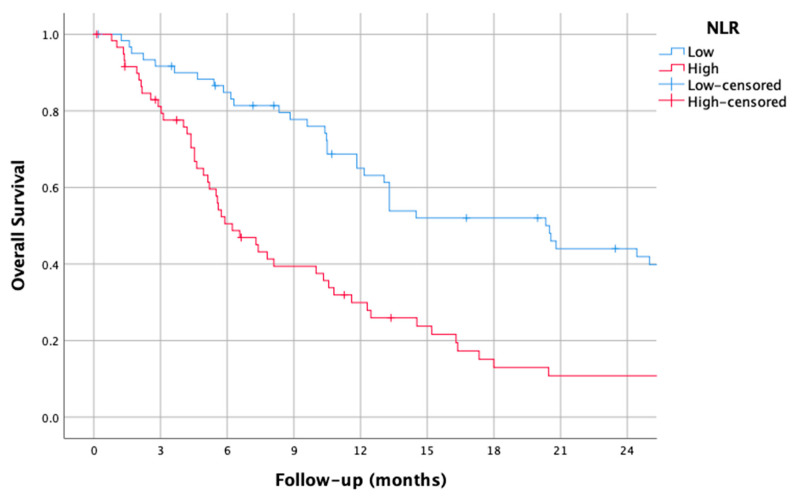
Kaplan–Meier overall survival curves for patients with high and low neutrophil-to-lymphocyte ratio (NLR).

The survival curves of the higher and lower NLR groups are shown in Figure 1.

### 3.2. PLR

The median PLR was found to be 184.1 (50.2–1204). Using this median as a cut-off, the median OS was 16.3 (8.02–24.57), H: 2.33 (1.54–3.52), *p* < 0.001 (95% CI).

**Figure 2:**
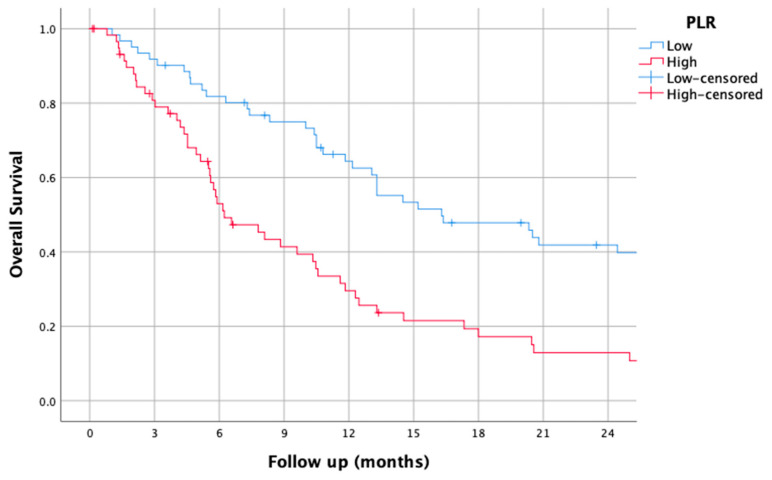
Kaplan–Meier overall survival curves for patients with high and low platelet-to-lymphocyte ratio (PLR).

The survival curves of the higher and lower PLR groups are shown in Figure 2.

### 3.3. LMR

The median LMR was 2.40 (0.58–15.0). Using this cut-off, the median OS of patients with a low LMR was 7.30 (5.05–9.55), while it was 15.2 (8.28–22.11) for those with a high LMR (HR of 0.6 (0.4–0.90), *p* = 0.014 (95% CI)).

**Figure 3:**
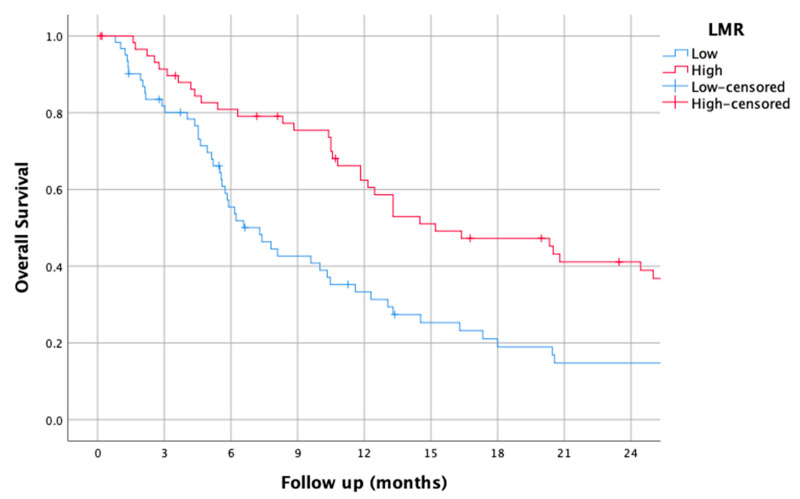
Kaplan–Meier overall survival curves for patients with high and low lymphocyte-to-monocyte ratio (LMR).

The survival curves of the higher and lower LMR groups are shown in Figure 3.

### 3.4. dNLR

The median dNLR was 0.012 (0–0.7). Using this cut-off, the median OS of patients with a low dNLR was 13.30 (5.14–21.46), while it was 7.30 (4.75–9.86) for those with a high dNLR (HR: 1.61 (1.08–2.43), *p* = 0.02).

**Figure 4:**
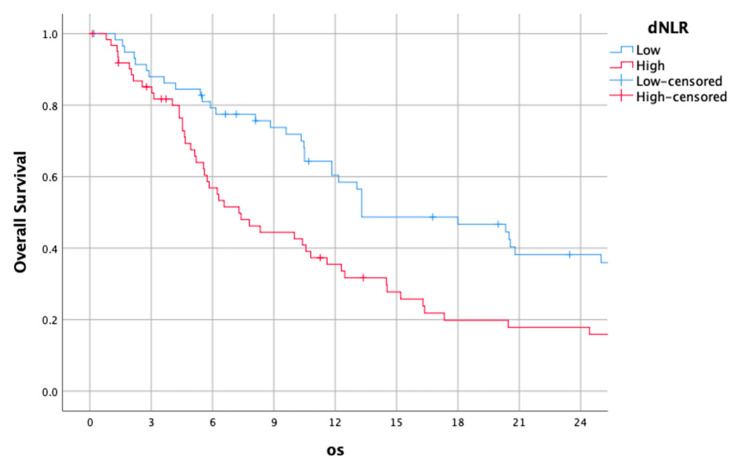
Kaplan–Meier overall survival curves for patients with high and low derived neutrophil-to-lymphocyte ratio (dNLR).

The survival curves of the higher and lower dNLR groups are shown in Figure 4.

### 3.5. SII

The median SII was 1024.82 (0–9641.0). Using this cut-off, the median OS of patients with a low dNLR was 20.5 (9.96–31.04), while it was 6.17 (4.14–8.2) for those with a high SII (HR of 3.16 (2.05–4.87), *p* < 0.001).

**Figure 5:**
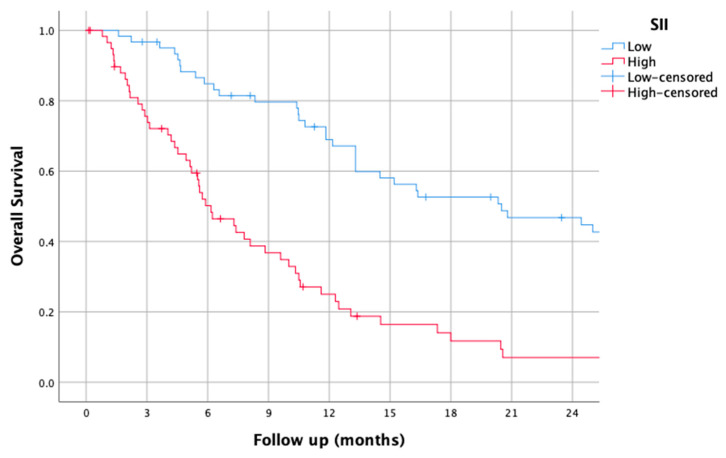
Kaplan–Meier overall survival curves for patients with high and low systemic immune-inflammation index (SII).

The survival curves of the higher and lower SII groups are shown in Figure 5.

### 3.6. CAR

The median CAR was 1024.82 (0–9641.0). Using this cut-off, the median OS of patients with a low CAR was 13.30 (9.17–17.43), while it was 5.73 (4.68–6.78) for those with a high CAR (HR of 2.07 (1.31–3.24), *p* = 0.002).

**Figure 6:**
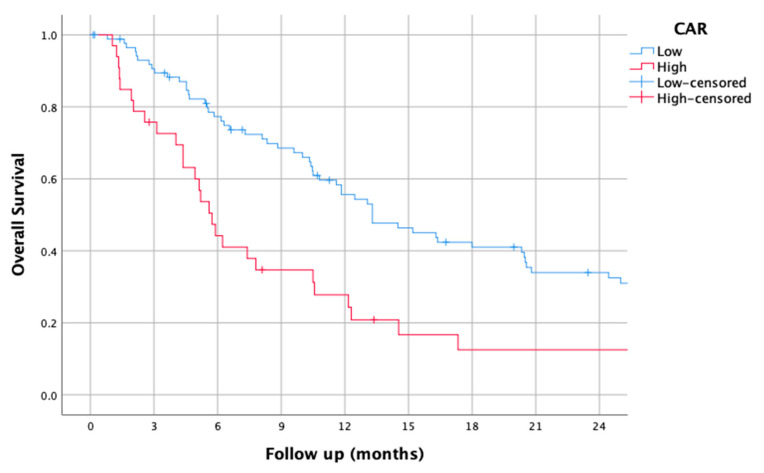
Kaplan–Meier overall survival curves for patients with high and low C-reactive protein-to-albumin ratio (CAR).

The survival curves of the higher and lower CAR groups are shown in Figure 6.

### 3.7. PNI

The median PNI was 169.86 (18.37–468.82). Using this cut-off, the median OS of patients with a low PNI was 6.30 (4.23–8.37), while it was 20.33 (12.30–28.36) for those with a high PNI (HR of 2.13 (1.4–3.23), *p* < 0.001).

**Figure 7:**
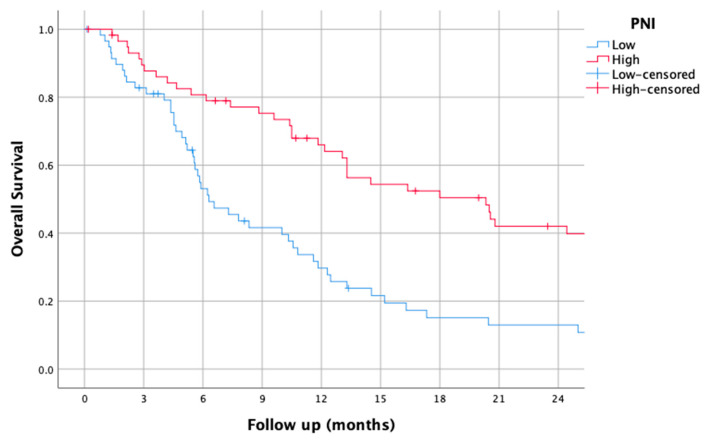
Kaplan–Meier overall survival curves for patients with high and low prognostic nutritional index (PNI).

The survival curves of the higher and lower PNI groups are shown in Figure 7.

### 3.8. mGPS

The mGPS was 0 for 29 patients (46.8%), 1 for 22 patients (35.5%), and 2 for 11 patients (17.7%). Using these groups, the median OS was 13.30 (7.18–19.42) in the 0 group, 5.60 (4.83–6.36) in the 1 group, and 3.13 (0.1–6.58) in the 2 group (HR of 2.2 (1.51–3.2), *p* < 0.01).

**Figure 8:**
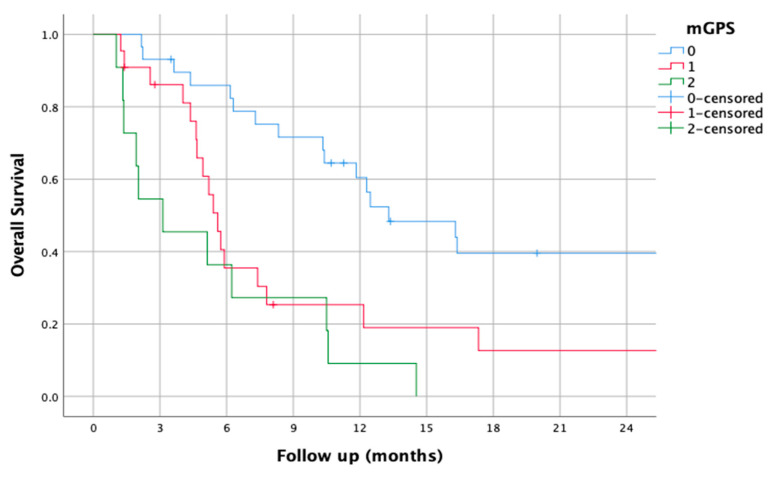
Kaplan–Meier overall survival curves for patients according to modified Glasgow Prognostic Score (mGPS).

The survival curves of the mGPS groups are shown in Figure 8.

### 3.9. PIV

The median PIV was 169.86 (18.37–468.82). Using this cut-off, the median OS of patients with a low PIV was 15.2 (9.0–21.4), while it was 7.4 (4.7–10.1) for those with a high PIV (HR of 1.76 (1.2–2.6), *p* < 0.001).

**Figure 9:**
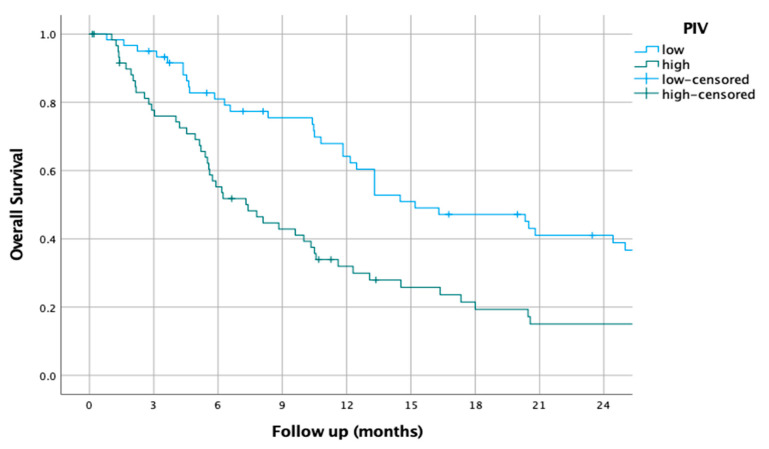
Kaplan–Meier overall survival curves for patients with high and low pan-immune inflammation value (PIV).

The survival curves of the higher and lower PIV groups are shown in Figure 9.

### 3.10. Bellmunt Score

The Bellmunt score was 0 for 66 patients (54.5%), 1 for 43 patients (35.5%), 2 for 11 patients (9.1%), and 3 for 1 patient (0.8%). Using these groups, the median OS was 16.30 (9.2–23.4) in the 0 group, 6.60 (4.6–8.5) in the 1 group, 10.0 (0.1–20.2) in the 2 group, and 4.93 in the 3 group (HR of 1.64 (1.24–2.18), *p* < 0.01).

**Figure 10:**
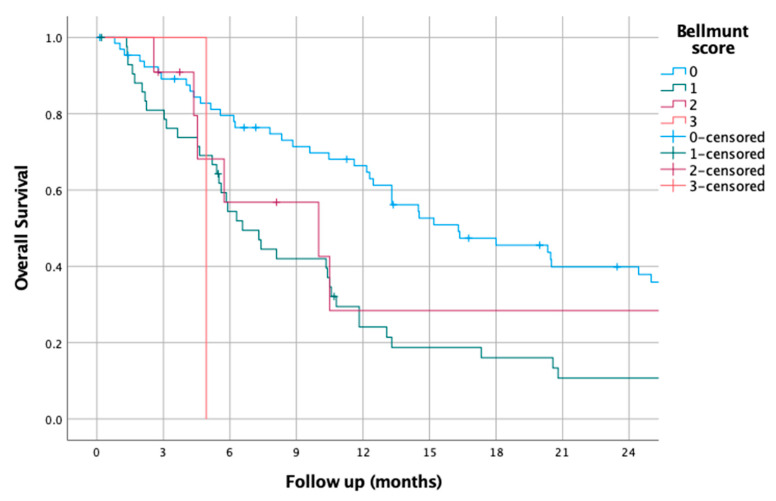
Kaplan–Meier overall survival curves for patients according to Bellmunt score.

The survival curves of the Bellmunt score groups are shown in Figure 10.

When performing a multivariate analysis of all blood-based biomarkers, the modified Glasgow Prognostic Score (mGPS) was the only biomarker that demonstrated a statistically significant association, with a hazard ratio (HR) of 1.984 (95% confidence interval [CI]: 1.16–3.4) and a *p*-value of 0.013.

The prognostic significance of various pretreatment blood-based biomarkers in metastatic bladder cancer was assessed using hazard ratios (HRs), 95% confidence intervals (CIs), and corresponding *p*-values (Table 2). The findings indicate that among the biomarkers analyzed, the modified Glasgow Prognostic Score (mGPS) demonstrated a statistically significant association with prognosis (HR = 1.984, 95% CI: 1.16–3.4, *p* = 0.013), suggesting its potential as a meaningful predictor of patient outcomes.

Other biomarkers, including the Bellmunt score (HR = 1.064, 95% CI: 0.71–1.6, *p* = 0.76), systemic immune-inflammation index (SII) (HR = 2.170, 95% CI: 0.55–8.6, *p* = 0.27), neutrophil-to-lymphocyte ratio (NLR) (HR = 1.530, 95% CI: 0.37–6.4, *p* = 0.56), and prognostic nutritional index (PNI) (HR = 1.001, 95% CI: 0.99–1.008, *p* = 0.87), did not reach statistical significance. Similarly, the platelet-to-lymphocyte ratio (PLR) (HR = 0.670, 95% CI: 0.23–1.95, *p* = 0.46), derived NLR (dNLR) (HR = 0.944, 95% CI: 0.34–2.6, *p* = 0.91), lymphocyte-to-monocyte ratio (LMR) (HR = 0.480, 95% CI: 0.18–1.29, *p* = 0.14), pan-immune inflammation value (PIV) (HR = 0.484, 95% CI: 0.16–1.5, *p* = 0.203), albumin-to-globulin ratio (AGR) (HR = 0.509, 95% CI: 0.2–1.15, *p* = 0.1), and C-reactive protein-to-albumin ratio (CAR) (HR = 0.603, 95% CI: 0.2–1.6, *p* = 0.32) did not show a statistically significant prognostic effect.

While most of these biomarkers did not exhibit independent prognostic significance, the findings highlight the potential utility of the mGPS in risk stratification for patients with metastatic bladder cancer. Further studies with larger cohorts are warranted to validate these results and explore their clinical implications.

Among all the evaluated variables, only the modified Glasgow Prognostic Score (mGPS) was identified as an independent prognostic factor for overall survival (HR = 1.846, *p* = 0.039), indicating that higher mGPS values are significantly associated with worse outcomes. Age also demonstrated a trend toward statistical significance (HR = 1.037, *p* = 0.054), suggesting a potential impact on survival, though it did not meet the predefined threshold for significance (*p* < 0.05).

When confounding factors were included in the additional analyses, the results remained consistent. Other biomarkers, including the Bellmunt score (HR = 1.103, *p* = 0.676), SII (HR = 2.668, *p* = 0.180), NLR (HR = 0.691, *p* = 0.629), and PNI (HR = 0.661, *p* = 0.525), did not reach statistical significance. Similarly, the PLR (HR = 0.463, *p* = 0.139), dNLR (HR = 0.963, *p* = 0.939), LMR (HR = 0.363, *p* = 0.067), PIV (HR = 0.546, *p* = 0.351), AGR (HR = 0.570, *p* = 0.178), and CAR (HR = 0.879, *p* = 0.819) did not demonstrate statistically significant prognostic value in the multivariate analysis.

## 4. Discussion

To the best of our knowledge, this is the first comprehensive investigation of a broad range of blood-based biomarkers within a single cohort of patients with mBC. Our study revealed significant associations between various pretreatment hematological biomarkers (NLR, dNLR, LMR, PLR, SII, mGPS, CAR, AGR, PNI, PIV, and Bellmunt score) and OS. However, when performing multivariate analysis, the mGPS emerged as the only biomarker demonstrating a statistically significant independent association with OS.

Previous studies have highlighted the prognostic significance of these biomarkers in various cancers. For instance, the NLR has been identified as a predictor of poor prognosis in several malignancies, including bladder cancer [15]. An elevated NLR is thought to reflect an enhanced inflammatory response, which can promote tumor progression and metastasis through various mechanisms, such as the suppression of immune surveillance and the promotion of a pro-tumorigenic environment [16]. Our findings align with these studies, as a higher NLR was associated with poorer OS in the univariate analysis.

The derived neutrophil-to-lymphocyte ratio (dNLR) also demonstrated significant prognostic value in our study. A higher dNLR was associated with worse OS, which is consistent with previous findings in other types of cancer, suggesting that this ratio can serve as a simple yet effective marker of systemic inflammation and immune response [8].

The PLR and LMR have been extensively studied in various cancers, including bladder cancer. A high PLR has been linked to poor prognosis, possibly due to the role of platelets in tumor growth [17]. Conversely, a low LMR, indicative of a lower lymphocyte count relative to monocytes, has been associated with worse outcomes, reflecting a weakened inflammatory response against the tumor [18]. Our study supports these findings, showing significant associations between these ratios and OS in univariate analyses.

The SII, which integrates the NLR and PLR into a single index, has recently gained attention as a prognostic marker in cancer [19]. A high SII indicates relatively elevated neutrophil and platelet counts, as well as reduced lymphocyte counts, which may suggest a strong pro-tumor inflammatory response and a compromised anti-tumor immune status [19]. Previous studies have shown that an elevated preoperative SII in bladder cancer patients undergoing radical cystectomy is independently associated with nodal invasion, advanced pT stage, and poor oncologic outcomes [20]. Higher SII levels were linked to shorter recurrence-free survival (RFS) and overall survival (OS), enhancing the predictive accuracy of existing prognostic models for locally advanced disease.

In our study, the SII did not retain independent prognostic significance in the multivariate analysis for metastatic bladder cancer. However, in the univariate analysis, a higher SII was significantly associated with worse survival. Further prospective studies and external validation are required to determine the prognostic value of the SII in the metastatic setting.

Among all the biomarkers analyzed, the mGPS was the only one that remained significant in the multivariate analysis, highlighting its strength as a prognostic indicator. Combining C-reactive protein (CRP) and albumin levels, the mGPS has been widely recognized as a measure of systemic inflammation and nutritional status in oncology. Its ability to capture both the inflammatory response and overall nutritional condition likely explains its strong prognostic value [21]. Although the mGPS does not influence standard treatment decisions, it carries potential clinical implications. Patients with a high mGPS are at increased risk of malnutrition and systemic inflammation, which suggests that nutritional support, muscle-preserving strategies, and targeted inflammation management should be integrated into their care. Moreover, patients with a high mGPS may potentially benefit from enrollment in clinical trials investigating new treatment options.

The prognostic nutritional index (PNI), derived from albumin levels and lymphocyte counts, has also been validated in various cancers, with lower PNI values indicating poorer outcomes [22,23]. Although the PNI was significant in the univariate analysis, it did not retain its significance in the multivariate model in our study, possibly due to the overlapping predictive capacity with other markers like the mGPS.

The Bellmunt score, which includes performance status, hemoglobin levels, and the presence of liver metastases as prognostic factors, is a well-established prognostic tool for mBC [24]. Our study confirmed its prognostic value in the univariate analysis, although it did not retain significance in the multivariate model. This could be due to the strong predictive power of the mGPS taking precedence over other markers.

Identifying reliable pretreatment biomarkers is essential for better assessing disease severity and tailoring treatment strategies in mBC. This study underscores the prognostic relevance of blood-based biomarkers, with the mGPS emerging as the most reliable independent predictor of OS. Moving forward, future research should focus on validating these findings in larger, prospective studies and incorporating systemic inflammatory markers into clinical decision-making. Additionally, emerging non-invasive biomarkers, such as urinary microbiome signatures, could provide further refinement in risk stratification [25]. Recent studies suggest that alterations in the urinary microbiome may contribute to bladder cancer progression, and potential interactions between microbial dysbiosis and systemic inflammation warrant further investigation. Combining systemic and localized inflammatory biomarkers in predictive models could enhance risk stratification, improve prognostic accuracy, and guide personalized treatment approaches for mBC. In conclusion, this study highlights the prognostic value of hematological biomarkers in metastatic bladder cancer. While multiple inflammatory markers offer insights into patient outcomes, the mGPS stands out as a particularly strong predictor of OS. Future research should aim to validate these findings prospectively and develop integrated prognostic models that incorporate both systemic and localized inflammatory biomarkers. A deeper understanding of these markers could ultimately lead to more precise survival predictions and improved therapeutic strategies for this aggressive disease.

## Figures and Tables

**Table 1:** Summary of clinical features and laboratory values of patients.

Variables	n or Median (Min–Max)
Age (years)	67.7 (39.71–93.9)
Age group	
<70	79 (60.39)
>70	52 (39.7)
Gender	
Female	17 (12.8)
Male	116 (87.2)
ECOG	
0–1	116 (87.9)
2–3	16 (12.1)
BMI	25.26 (17.17–41.4)
WBC	8.2 (3.4–22.4)
Hgb	11.9 (7.2–16.4)
Lymphocytes	1.6 (0.1–4.6)
Neutrophils	5.4 (2.1–20.8)
Monocytes	0.6 (0.1–1.65)
Platelets	283 (92–635)
Albumin	3.8 (1.99–4.79)
Globulin	3.28 (1.77–4.71)
CRP	3.47 (0.15–46.3)
PFS	5.98 (0.1–35.6)
OS	10.4 (0.13–89.4)
Deceased	
No	29 (21.8)
Yes	104 (78.2)

**Table 2:** Multivariate analysis of all blood-based biomarkers.

Biomarker	HR	95% CI for HR	*p*-Value
Bellmunt score	1.064	0.71–1.6	0.76
SII	2.170	0.55–8.6	0.27
PLR	0.670	0.23–1.95	0.46
NLR	1.530	0.37–6.4	0.56
dNLR	0.944	0.34–2.6	0.91
LMR	0.480	0.18–1.29	0.14
PIV	0.484	0.16–1.5	0.203
AGR	0.509	0.2–1.15	0.1
CAR	0.603	0.2–1.6	0.32
mGPS	1.984	1.16–3.4	**0.013**
PNI	1.001	0.99–1.008	0.87

## Data Availability

The data sets generated and/or analyzed during the current study are available from the corresponding author upon reasonable request.
